# Analysis of Mitochondrial 3D-Deformation in Cardiomyocytes during Active Contraction Reveals Passive Structural Anisotropy of Orthogonal Short Axes

**DOI:** 10.1371/journal.pone.0021985

**Published:** 2011-07-11

**Authors:** Yael Yaniv, Magdalena Juhaszova, Su Wang, Kenneth W. Fishbein, Dmitry B. Zorov, Steven J. Sollott

**Affiliations:** 1 Laboratory of Cardiovascular Science, Gerontology Research Center, Intramural Research Program, National Institute on Aging, National Institutes of Health, Baltimore, Maryland, United States of America; 2 Laboratory of Clinical Investigation, Gerontology Research Center, Intramural Research Program, National Institute on Aging, National Institutes of Health, Baltimore, Maryland, United States of America; Université Joseph Fourier, France

## Abstract

The cardiomyocyte cytoskeleton, composed of rigid and elastic elements, maintains the isolated cell in an elongated cylindrical shape with an elliptical cross-section, even during contraction-relaxation cycles. Cardiomyocyte mitochondria are micron-sized, fluid-filled passive spheres distributed throughout the cell in a crystal-like lattice, arranged in pairs sandwiched between the sarcomere contractile machinery, both longitudinally and radially. Their shape represents the extant 3-dimensional (3D) force-balance. We developed a novel method to examine mitochondrial 3D-deformation in response to contraction and relaxation to understand how dynamic forces are balanced inside cardiomyocytes. The variation in transmitted light intensity induced by the periodic lattice of myofilaments alternating with mitochondrial rows can be analyzed by Fourier transformation along a given cardiomyocyte axis to measure mitochondrial deformation along that axis. This technique enables precise detection of changes in dimension of ∼1% in ∼1 µm (long-axis) structures with 8 ms time-resolution. During active contraction (1 Hz stimulation), mitochondria deform along the length- and width-axes of the cell with similar deformation kinetics in both sarcomere and mitochondrial structures. However, significant deformation anisotropy (without hysteresis) was observed between the orthogonal short-axes (i.e., width and depth) of *mitochondria* during electrical stimulation. The same degree of deformation anisotropy was also found between the *myocyte* orthogonal short-axes during electrical stimulation. Therefore, the deformation of the mitochondria reflects the overall deformation of the cell, and the apparent stiffness and stress/strain characteristics of the cytoskeleton differ appreciably between the two cardiomyocyte orthogonal short-axes. This method may be applied to obtaining a better understanding of the dynamic force-balance inside cardiomyocytes and of changes in the spatial stiffness characteristics of the cytoskeleton that may accompany aging or pathological conditions.

## Introduction

The cardiomyocyte cytoskeleton is composed of rigid and elastic elements and maintains the shape of the cell as an elongated cylinder with an elliptical cross-section, even during contraction-relaxation cycles. During contraction of the heart, most of the work performed during active myocyte shortening is external and goes into the dynamic ejection of blood from the ventricle (i.e., pressure-volume work), while a smaller, but still significant fraction is transiently stored as potential energy (PE) inside the cell by the strain of internal elastic elements of the cytoskeleton. This PE stored in the cytoskeleton, harnessed during relaxation, may contribute significantly to the relaxation of the ventricle and therefore affect the efficient filling of the heart with blood [1].

Cardiomyocyte mitochondria are micron-sized fluid-filled passive spheres distributed throughout the cell in a crystal-like lattice and occupy ∼35% of the myocyte volume [2]. They are individually situated in pairs sandwiched between the contractile machinery of the sarcomere, longitudinally and radially; thus, their shape represents the three-dimensional balance of forces extant at any given moment. While regulated mitochondrial matrix volume changes can occur over a relatively slow time frame [3], [4], these organelles can be considered to be essentially isovolumic on the time-scale of active contraction-relaxation.

The mitochondria, being essentially passive structures in the cell, can serve as a model Eulerian system whereby analysis of their deformations in response to cell contraction and relaxation can provide detailed insights into the active and passive stress-strain characteristics of the enclosing cell as well as into tissue behavior. Moreover, the question arises whether forces exerted on mitochondria during myofilament contraction and relaxation might modulate the intrinsic function of those organelles (e.g., by membrane mechanosensitive processes, etc.). It is also possible that in response to a substantial increase in the myofilament strain (due to muscle stretch), the mitochondria might be induced to change volume to regulate their function. For example, it has been proposed that changes in mitochondrial Ca^2+^ concentration in response to increases in myofilament strain can induce changes in mitochondrial volume that are accompanied by an increase in respiration [5]. These processes have never been examined in detail. By developing a novel method to examine the average deformation of mitochondria in three dimensions in response to cardiomyocyte contraction and relaxation we sought to understand how dynamic forces are borne and balanced inside the cardiomyocytes.

We previously described a method to simultaneously measure sarcomere and mitochondrial dimensions *in situ* along the long-axis of quiescent cardiomyocytes [3]. This technique is based on the variation in transmitted light intensity induced by the periodic lattice of myofilaments alternating with rows of mitochondria. This spatial variation in optical signal is analyzed by examining the amplitude of the 1^st^ and 2^nd^ order peaks in the Fourier transform frequency spectrum of the transmitted light intensity along a given cardiomyocyte axis. Using this method, we have shown that certain signals that modulate the mitochondrial diameter by ∼1–1.5% significantly change the mitochondrial function under these conditions [3]. Here we extend this method to quantify the average sarcomere length (SL) and mitochondrial dimensions along all three axes during the contraction-relaxation cycle.

**Figure 1:**
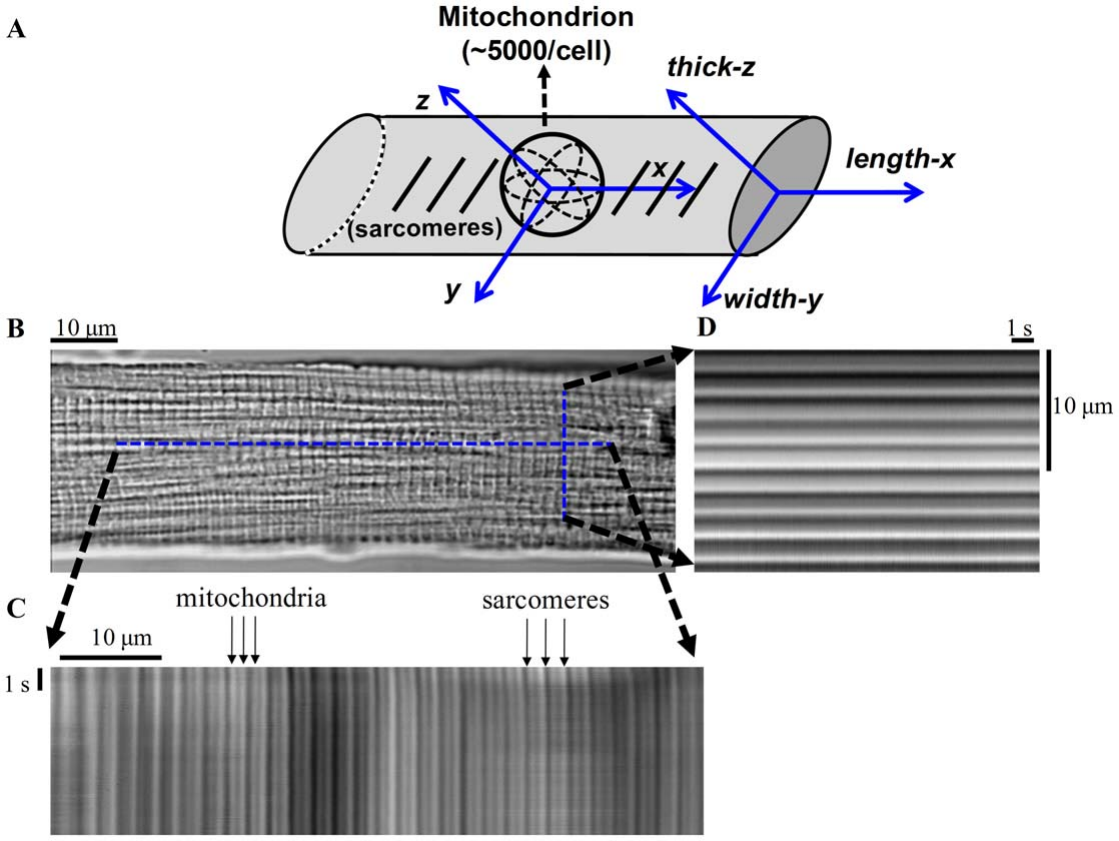
Isolated cardiomyocyte geometry with *in situ* mitochondria and sarcomere structures. (A) Schematic diagram of the coordinate system (X-Y-Z axes) of isolated myocytes and *in situ* mitochondria. (B) Brightfield image of cardiac myocyte showing orientation of laser line-scan imaging of in situ mitochondria along the orthogonal (C) *length-x* and (D) *width-y* axes of the cell. The cell structure, consisting of sarcomeres (∼1.9 µm structures between dark z-lines) and mitochondria (∼0.95 µm structures) is evident.

Quantitative analysis of mitochondrial and sarcomere deformation in intact cardiomyocytes during active contraction (1 Hz stimulation frequency) with 8 ms time resolution and 10 nm (1% over 1 µm) spatial resolution revealed measurable compression of the mitochondria along the long-axis (see definitions in Figure 1A) with similar time-to-peak deformation and 50% and 90% relaxation kinetics in both sarcomere and mitochondrial structures. We validated the short-axis measurements by performing Fourier spectral analysis of cells loaded with tetramethylrhodamine methyl ester (TMRM), a dye that provides high-contrast vital staining of healthy mitochondria, and by exposing the cells to a mitochondrial K_ATP_ (mitoK_ATP_) channel opener that induces a small degree of mitochondrial swelling without causing detectable changes in the sarcomere length [3]. Along the short-axis, this analysis revealed mitochondrial expansion during cell contraction with similar deformation kinetics as was observed along the long axis. We found that exposure to the mitoK_ATP_ channel opener diazoxide (Dz) induced mitochondrial volume changes on the order of 2-4%, in accordance with reported long-axis swelling in quiescent cardiomyocytes [3] and in suspensions of isolated mitochondria [6], [7].

Under the assumption of constant mitochondrial volume, unequal deformation was observed along the mitochondrial short-axes during electrical stimulation. Interestingly, the same degree of deformation anisotropy occurred between the short axes of the myocyte during the same electrical stimulation. Therefore, the mitochondrial deformations reflect the overall deformations of the cell (consistent with an Eulerian model system), and the stiffness and stress-strain characteristics of the cytoskeleton are found to be unexpectedly distinct between the two short-axes. This analytical method may allow a deeper understanding of the dynamic force-balance inside the cardiomyocytes and of potential changes in the spatial stiffness characteristics of the cytoskeleton during aging, as well as after damage to the heart. This could potentially lead to new insights regarding the nature of the impaired diastolic properties of the heart under these conditions, as well as to the function of mitochondria and the regulation of energy supply-demand matching.

## Results

### Cardiomyocyte structural determinants of principal Fourier transform spectral peaks: assignment and validation

**Figure 2:**
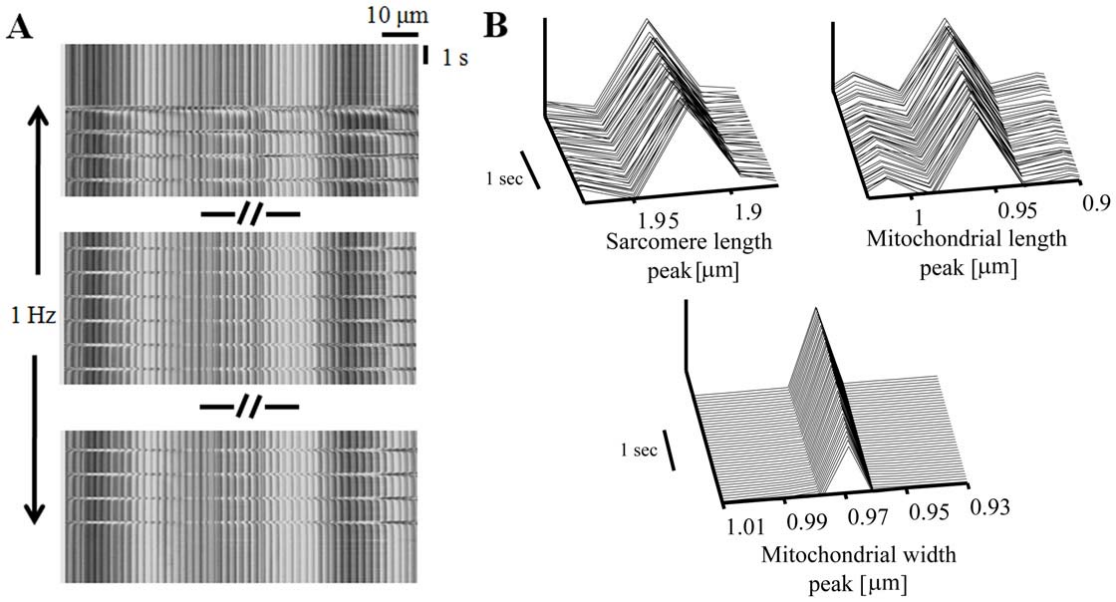
Fourier transform spectra obtained from linescan imaging to determine sarcomere and mitochondrial dimensions. (A) Transmitted optics line-scan image along cardiomyocyte long-axis (*length-x*) during rest and electrical stimulation (1 Hz). (B) Sarcomere length and mitochondrial length and width peaks during quiescent state determined from frequency domain analysis.

We previously described a protocol to simultaneously measure sarcomere and mitochondria dimensions along the long-axis of quiescent cardiomyocytes [3]. Here we extend this method to also measure 1) the mitochondrial dimension along the width-axis (Fig. 2B, lower panel), and, 2) sarcomere and mitochondrial dimensions during both quiescence and electrical stimulation.

Two different approaches that we have described previously [3] were utilized here to prove that the power of harmonics contributed by certain birefringent bands of the sarcomere does not significantly interfere with information independently associated with the mitochondrial diameter along the length-axis, and to confirm that the appropriate peak along the width-axis also corresponds to the mitochondrial dimension along this axis. The first approach utilizes confocal imaging of cells loaded with the fluorescent dye, TMRM, which sequesters inside mitochondria to achieve a concentration several orders of magnitude greater than in other cellular compartments, providing a high contrast mitochondria-selective fluorescence image. The second approach relies on transmitted-light imaging of control cells compared to those exposed to the mitoK_ATP_ channel opener, Dz, which induces mitochondrial swelling [3], [6] without affecting the SL [3].

**Figure 3:**
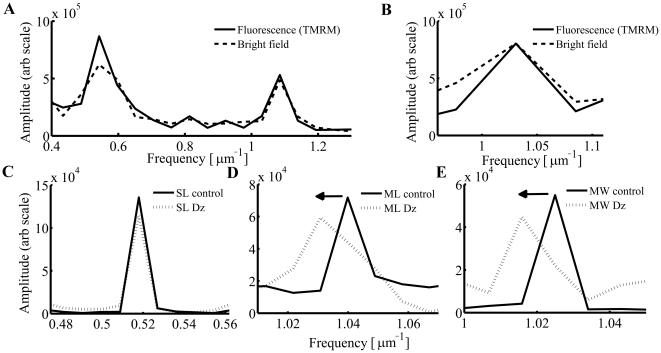
Cardiomyocyte structural determinants of principal Fourier transform spectral peaks: assignment and validation. Consecutive Fourier analysis along length (A) and width (B) directions calculated from bright field and fluorescence images of tetramethylrhodamine methyl ester (TMRM) loaded cardiomyocytes. Low concentrations of diazoxide (Dz; 30 µM) did not shift the sarcomere peak (C), but shifted the frequency of the mitochondrial peak (D) along the length-axis and (E) width-axis to the left by ∼1%.

We applied Fourier spectral analysis to X-Y frame-scanned confocal images of TMRM-loaded cardiac myocytes. To avoid potential complications arising from the production of reactive oxygen species due to the excitation of TMRM by 543 nm laser irradiation during standard fluorescence imaging, which could lead to induction of the mitochondrial permeability transition pore [8], these images were acquired only after all transmitted optics line-scans (obtained using a 633 nm laser) were collected. These fluorescence image scans provided high-contrast images of the periodic lattice of mitochondria arrayed within sarcomeres along the length-axis as well as the periodic lattice of mitochondria arrayed along the width-axis, without significant contribution from non-mitochondrial structures. Figures 3A and 3B show the pattern and resolution of length and width peaks obtained from fluorescence images and their correlation with the transmitted optics line-scan peaks obtained from the same cell.

As has been demonstrated [3], exposure to low concentrations of Dz (30 µM) shifts the mitochondrial peak to lower frequency along the length-axis (Fig. 3D), signifying an increase of the mitochondrial diameter along this axis by ∼1% without any detectable shift in the sarcomere peak (Fig. 3C). This observation rules out any significant birefringent interference of the principal sarcomere structures with the signal from mitochondrial structures along the length-axis. In order to confirm that our inability to detect changes in SL due to Dz treatment was not due to the spatial resolution employed, we further increased (doubled) the spatial resolution by to allow precise resolution of changes down to 19.5 nm (1% of a 1.95 µm long structure). Again, no changes in SL after exposure to Dz were found using this technique (Fig. 3C). Additionally, Dz shifted the mitochondrial peak along the width-axis toward lower frequency (Fig. 3E), indicating that the mitochondrial width increased by the same amount as the mitochondrial length. This is consistent with a small (∼4%) volume increase, indicating again that the peak analyzed did indeed correspond to mitochondrial width and not to the dimension of another cellular structure.

### Deformation of mitochondria in response to sarcomere contraction

**Figure 4:**
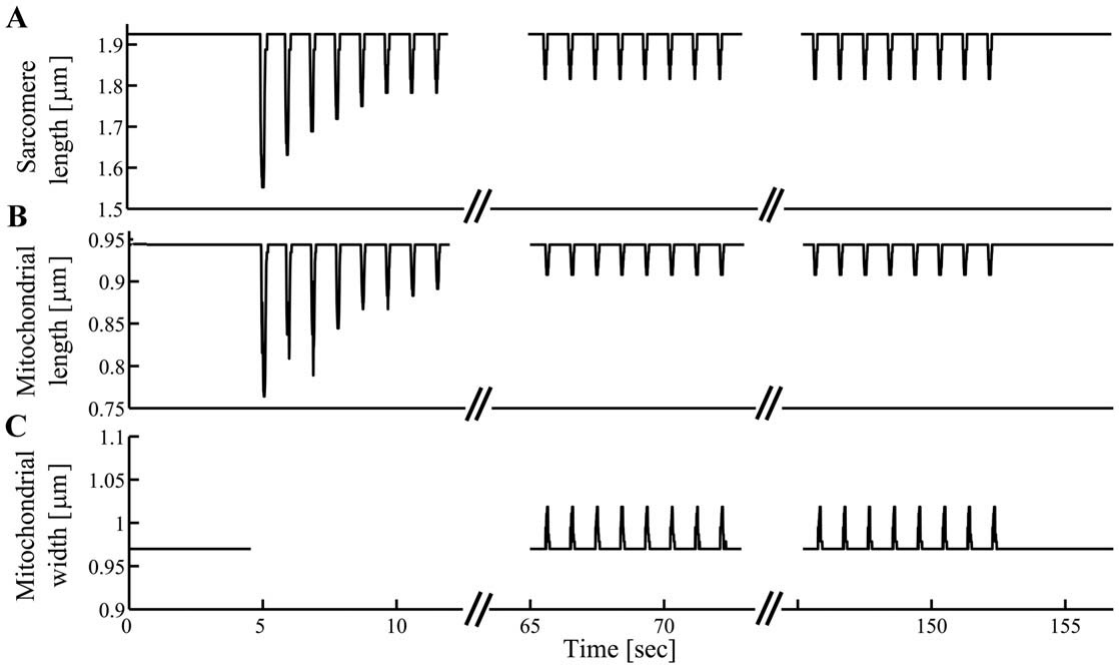
Deformation of mitochondria in response to sarcomere contraction. (A) Sarcomere length and (B) mitochondrial length calculated from frequency domain analysis along *length-x* axis during electrical stimulation (1 Hz). (C) Mitochondrial width calculated from frequency domain analysis along *width-y* axis during electrical stimulation (1 Hz).

Figure 4A presents the SL during quiescence, the contractile staircase from rest, and steady state contractions, derived from the period of the first (lower frequency) major peak. Figure 4B shows the mitochondrial length during the same intervals, derived from the period of the second (higher frequency) peak. Along the length-axis, both structures shortened (compressed) during contraction. The principal contractile parameters (Table 1) of the SL and mitochondrial diameter along the length-axis were the same, except for the extent of deformation and resting dimensions. Note that the two-fold difference in these parameters derives solely from the innate structure of the cardiomyocyte, which typically has exactly two mitochondria per sarcomere. The sarcomere contraction parameters measured under control conditions are in full accordance with those published in other studies (e.g., [9], [10]). Figure 4C shows the deformation of mitochondrial width, which increased during cardiomyocyte contractions. The time-to-peak deformation, and 50% and 90% relaxation were similar for mitochondrial width and length. The differences in extent-of-deformation and fractional-deformation between the width- and thick-axes are interpreted as the degree anisotropy between those respective axes.

**Table 1:** Sarcomere and mitochondria deformation parameters.

	Sarcomere Length (n = 17)	Mitochondrial-Length (n = 17)	Mitochondrial-Width (n = 17)
**Extent of deformation (µm)**	−0.16±0.01	−0.08±0.01*	0.06±0.01*
**Fractional deformation (%)**	−8.7±0.7	-8.3±0.7	6.2±0.3*
**Time to peak deformation (ms)**	54±3	63±5	59±4
**Deformation duration to point of 50% relaxation (ms)**	124±6	122±6	110±7
**Deformation duration to point of 90% relaxation (ms)**	162±8	159±8	152±7
**Rest dimension (µm)**	1.92±0.01	0.95±0.01*	0.98±0.01* ^,^ **

All data are presented as mean +/− SEM,

*P<0.05 vs. sarcomere,

**P<0.05 vs. mitochondrial-length.

### Change in “diastolic” mitochondrial dimension in response to sarcomere contraction

It was shown in suspensions of isolated mitochondria that certain small changes in Ca^2+^ concentration can induce small and reversible changes in mitochondrial volume along with a parallel increase in respiration [5]. Based on this finding, it was postulated [11], [12] that an increase in mitochondrial Ca^2+^, which might be naturally achieved via electrical stimulation of cardiomyocytes, could also lead to an increase in mitochondrial volume *in situ*. We have shown here (see Figs. 4B and 4C for a representative example) that there are no significant changes in the “diastolic” mitochondrial dimensions (i.e., in the length- and width-axes during relaxation between the cyclic deformation caused by contractions) during steady-state 1 Hz electrical stimulation compared to quiescence. Therefore, in mechanically unloaded intact cells during light pacing work, there is no detectable change in mitochondrial volume accompanying any small pacing-related increase in mitochondrial Ca^2+^.

### Anisotropic deformations of *in situ* cardiomyocyte mitochondria

**Figure 5:**
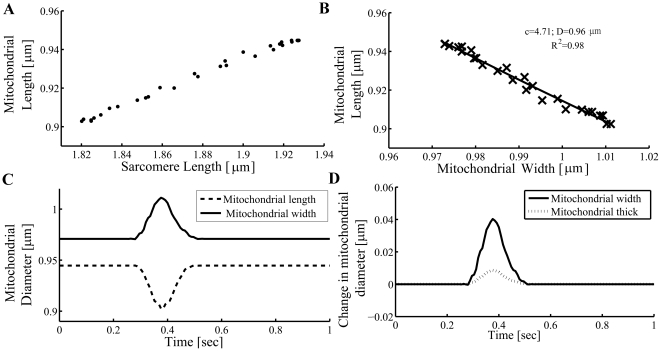
Sarcomere-mitochondrial length and mitochondrial length-width relationships during cardiomyocyte contraction-relaxation cycle. The relationship between mitochondrial length and (A) sarcomere length or (B) mitochondrial width. ***c*** is the coefficient of anisotropy (ratio of deformation magnitude between the *width-y* and *thick-z* axes) and *D* is the average mitochondrial diameter. (C) Mitochondrial length and width vs. time during a single contraction cycle. (D) Change in mitochondrial width and calculated thickness vs. time during a single contraction cycle. Data shown is from a representative single cardiomyocyte experiment.

Figure 5A represents the SL vs. the mitochondrial dimension in the length-axis. In order to estimate the deformation of the mitochondrial dimension in the thick-axis, we assumed constant mitochondrial volume during a single contraction transient and ellipsoidal (nearly spherical) geometry. Mitochondrial volume (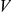) can be calculated according to:
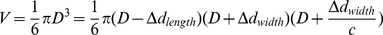
(1)where *D* is the average mitochondrial diameter (i.e., that of the equivalent-volume sphere), 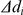
is the change in diameter due to the deformation along the respective orthogonal reference-axis, and *c* is the “coefficient of anisotropy” (ratio of deformation magnitude between the width- and thick-axes; when *c* = 1, there is no anisotropy). Rearrangement of equation 1 yields the constant-volume approximation relationship between mitochondrial deformation in the width- and in the length-axes.
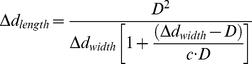
(2)


Figures 5B and C show a representative example of the relationship between the mitochondrial dimensions along the length- vs. width-axes during 1 Hz steady-state cardiomyocyte contraction. The tight fit (R^2^ = 0.93; n = 14, *P*<0.001) in the ensemble data set between the experimental data and the constant-volume approximation is consistent with this assumption being valid. The average mitochondrial equivalent-sphere diameter, *D*, was 0.97±0.01 µm. The *c* coefficient (ensemble) obtained was 3.7±0.3, which, being far from 1, indicates that there also might be significant passive structural anisotropy along the orthogonal short-axes of the cardiomyocyte. Measurement of this anisotropy of mitochondrial deformation allowed us to calculate the deformation (change) of mitochondrial dimension in the thick-axis during contraction (see representative example in Fig. 5D).

### The anisotropic deformability of the *in vitro* cardiomyocyte

Cardiac mitochondria have a near-spherical shape when isolated [4], [13], and have an essentially circular cross-section inside quiescent cardiomyocytes (present results). Furthermore, since Dz, by a small regulatory expansion of mitochondrial matrix volume (∼2–4%), causes a symmetric expansion in mitochondrial dimension (i.e., along the length- and width-axes; present results), it is reasonable to assume that the bulk mechanical properties of mitochondria are essentially isotropic (i.e., independent of direction in space) and that their internal stress-strain characteristics contribute negligibly to the bulk properties of the surrounding cardiomyocyte cytoarchitecture, so that assessment of directional deformations may be interpreted in light of the forces and material properties of the surrounding environment (i.e., that of the interior of the cardiomyocyte). Thus, we conclude that mitochondria behave as micro-scale “test balloons” that can be used to reflect the stress-strain properties of their environment. Next, we evaluated the corresponding deformations of the cell along each axis in order to determine whether a significant passive structural anisotropy exists within the cell. We assumed constant cell volume during a single contraction transient and cylindrical geometry with an elliptical cross-section having a thickness to width ratio of one to three, which closely approximates intact cell dimensions [14]. Based on these assumptions cell volume (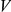) can be calculated according to:
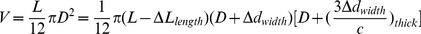
(3)where *L* is the cell length, 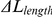
is the change in cell length due to the deformation during cardiomyocyte contraction, *D* is the quiescent cardiomyocyte width, 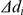 is the change in diameter due to the deformation of the respective orthogonal reference-axis, and *c* is the coefficient of anisotropy. Rearrangement of equation 3 yields,
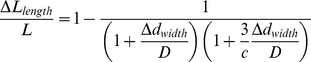
(4)


**Figure 6:**
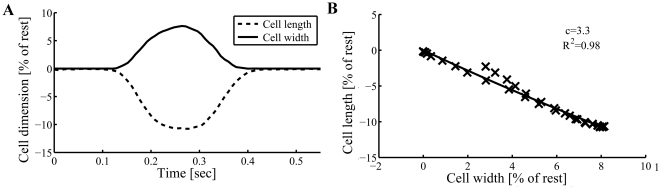
Cell length-width relationship during cardiomyocyte contraction-relaxation cycle. (A) The percent change in the cell-length and cell-width vs. time during a single contraction cycle. (B) The measured relationship between percent change in cell-length vs. percent change in cell-width. ***c*** is the coefficient of anisotropy (ratio of deformation magnitude between the *width-y* and *thick-z* axes). Data shown is from a representative single cardiomyocyte experiment.

Figure 6A shows a representative plot of the cell length vs. the cell width during 1 Hz steady-state contraction. Note that the cell deformed along the length- and width-axes in a qualitatively similar fashion to the mitochondrial deformation along the same axes (Fig. 5C); the cell shortened in the length axis and expanded in the width axis. The tight fit (R^2^ = 0.93; n = 13, *P*<0.001) between the ensemble experimental data set and the constant volume approximation is consistent with the model assumptions being valid (see Fig. 6B). The *c* coefficient (ensemble) obtained was 3.0±0.4, similar to that seen in mitochondria, which confirms that there is significant passive structural anisotropy along the cardiomyocyte short-axes as we observed in mitochondria.

## Discussion

Mitochondrial volume regulation can impact and control ionic homeostasis (see [15], [16] for recent reviews) and activate signals involved in cell protection [3]. Because *in situ* mitochondria can behave differently from those in isolated suspension, accurate and reproducible measurements of *in situ* mitochondrial properties, including their dimensions, are *de rigueur* to explore their functions in the native structural and signaling environment. The present study is the first, to our knowledge, that describes *in situ* 3D measurements of mitochondrial and cardiomyocyte deformations during contraction-relaxation cycling. These measurements reveal similar anisotropic deformations along the orthogonal short axes in both mitochondria and cardiomyocytes consistent with structural anisotropy within the cytoskeleton. We will discuss the context, significance and potential implications for mitochondrial function of these findings as well as interpret them with respect to cardiomyocyte and ventricular function.

### Existing approaches to assess changes in mitochondrial size, shape or volume

An ensemble average mitochondrial volume has been indirectly measured by several groups under *in vitro* conditions: the Halestrap [5], [17], Garlid [6], and Weiss [7] laboratories have estimated the steady-state matrix volume in an isolated mitochondrial suspension by measuring light scattering at 520 nm, which has been found to vary linearly with matrix volume. A major limitation associated with this technique is the artificial situation created when the mitochondria are removed from the cells, i.e., their removal from the normal cytoskeleton architecture and cytosolic signaling components and other relevant aspects of the intracellular milieu (not to mention unforeseen effects of mechanical-chemical damage to the organelle due to isolation *per se*). Therefore, factors that regulate mitochondrial volume in suspensions of isolated mitochondria may not operate as they would under *in situ* conditions [3], [13], [18].

A 3D electron microscopic tomography method reveals the cell structures in heart tissue with high resolution [19]. However, this method does not allow the measurement of dynamic changes in mitochondrial dimension. Furthermore, artifacts of dehydration and fixation, which differentially affect adjacent cellular structures, e.g., myofilaments and mitochondria, might lead to one component being relatively stiffer and incompressible or even excessively strained relative to its neighbor, resulting in unnatural compression or elongation of mitochondria between myofilaments.

Veksler's group [20], [21] has developed a fluorescence method (i.e., using membrane potential-insensitive dye MitoTracker Green) to measure the mitochondrial volume in permeabilized fibers and cells. In this method the mitochondrial dimensions are measured directly, from each edge, with the detection resolution limited by the optical microscope diffraction limit and by the ambiguity in detecting precisely the mitochondrial edges from fluorescence pictures (due to inherent blurring). Our method allows precise quantification of the mitochondrial dimensions by taking advantage of the crystal-like order of the organelle's structure and distribution, enabling resolution of a 10 nm *change* in a 1 µm-long structure (Fig. 2) specifically without unwanted photochemical effects (see above). Additionally, when permeabilized cells are treated with pharmacological agents (which can ordinarily induce just a small degree of mitochondrial swelling when applied to intact cells [3]), mitochondria may unnaturally expand their volumes until they can mechanically compress the myofilament structure, and, in turn impact development of contractile force and presumably also affect the resting SL [22]. Note that Veksler's group reports a 60% increase in mitochondrial volume by treatment with propranolol [21] which is more than an order of magnitude higher that we estimated for treatment with Dz (Fig. 3). This is probably unlikely to be a physiological change observed *in situ*. Under our conditions in the present work, we did not detect changes in the SL or contractile parameters with Dz (data not shown). Indeed, our novel *in situ* approach allows examination of regulatory volume changes in intact mitochondria (due to signaling) independent of the stress-strain characteristics of the myofilaments and contractile dynamics ([3] and present results). Generally, permeabilized cells (although useful) are inferior physiological models compared to intact cells for the exploration of mitochondrial function akin to the whole heart. This is illustrated, for example, by the markedly different sensitivities to Ca^2+^-induction of the permeability transition pore inside intact cardiomyocytes vs. that in permeabilized cells or in suspensions of isolated mitochondria [3], [18].

### Interpretation of *in situ* mitochondrial deformation during pacing work conditions

In suspensions of isolated cardiac mitochondria, several regulators were shown to induce mitochondrial swelling including: osmotic strength [17], mitoK_ATP_ channel openers such as Dz [6], [7], and Ca^2+^ concentrations in the mitochondria [5]. Because *in situ* mitochondria can behave differently than when isolated in suspension, the question arises regarding whether such changes occur under *in situ* conditions. We have previously shown that different mitoK_ATP_ channel openers can increase the mitochondrial length-axis dimension by ∼1% in intact cardiomyocytes [3]; this is apparently a much smaller change that that observed in suspensions of isolated mitochondria. We have extended this result here, finding that mitochondrial width and length dimensions increase to the same degree when such changes are up to 10 nm (Fig. 3). However, the relative effect of Ca^2+^-concentration on mitochondrial volume appeared to differ between the *in situ* and *in vitro* models. We have shown here (Fig. 4) that there is no significant difference in the “diastolic” mitochondrial length- or width-dimension during light pacing work compared to resting conditions. Increase in mitochondrial volume is associated with an increase in respiration in both *in situ* and *in vitro* models [3], [23], where such a respiratory change can serve to facilitate matching ATP supply to demand. However, the increase in cytosolic Ca^2+^ due to Ca^2+^ cycling during light pacing work did not detectably increase mitochondrial volume, and thus we infer that volume-activated Ca^2+^-transport into mitochondria is unlikely to play a key role in matching ATP supply and demand at low workloads. It was hypothesized that pressure or conformation changes in the mitochondrial cristae structure might augment the drive for ATP synthesis by F_1_F_o_-ATP synthase [24], [25]. Moreover, the question arises whether the cell deformations can be sensed by the mitochondria (e.g., by mechanosensitive structures/channels on the membrane [26] which sense changes in membrane stress and/or curvature) and thereby result in adaptive changes in function, such as to facilitate an increase in ATP production to meet an increase in cellular demand. Our work may allow estimation of the *in situ* pressure gradients, and changes in membrane curvature and stress, to evaluate whether such mechanisms might be signaling mitochondria to match ATP supply to demand.

### Cytoskeleton and myofilament properties based on mitochondrial 3D deformations

We show here, for the first time, how the mitochondria deform in three dimensions during cardiomyocyte contraction and relaxation. Since the mitochondria are distributed between, and are in dynamic force balance with, the cytoskeletal proteins [27] at various levels of tension, it is fruitful to analyze how mitochondrial deformations reflect the surrounding behavior of the cardiomyocyte. We found that the mitochondria expanded asymmetrically along the width- and thick-axes during cell contraction (Fig. 5B, D). Similarly, the cardiomyocyte body expanded along the same axes during contraction, which is in accordance with Boyett et al. [14], with a coefficient of anisotropy, *c*, comparable to that of the mitochondria (see Results). We saw this as an opportunity to analyze and extract the nature of certain passive tension-bearing elements of the cytoskeleton that, as the cell shortens, store a fraction of the energy of contraction as PE that can be recovered upon relaxation (and indeed is responsible for the “restoring force” that in part governs relaxation in the isolated cardiomyocyte model). There are several main considerations and assumptions that directly bear on this discussion:

1. Mitochondria across a wide variety of matrix volumes and isolation artifacts adopt a near-spherical shape in isolation [4], [13].
2. Mitochondria with physiological matrix volumes have a circular cross-section (along length and width) inside quiescent cardiomyocytes (present results).
3. Small regulatory matrix volume increases (e.g., induced by Dz) result in symmetrical increases in that circular cross-section (present results). Thus, based on points 1–3, whatever internal structural components there may be inside mitochondria, their internal stress-strain characteristics are negligible on the scale of those forces/pressures/etc. within the interior of the cardiomyocyte under loading/compression during contraction/relaxation mechanical cycles.
4. When mitochondria inside cardiomyocytes are compressed, we assume that the internal pressure of each mitochondrion increases as if it were a single compartment, and that small deformations from its spherical shape, measured along the respective orthogonal axes, are accompanied by effectively equivalent areas of surface contact with adjoining structures so that we can consider each of these vector forces to be effectively equivalent.
5. Finally, for small deformations, we can assume approximately Hookean-like properties, at least for qualitative discussion purposes.

With these assumptions we can conclude that for small deformations, mitochondria respond to their environment without substantially affecting the stress-strain characteristics of that environment. That the deformation anisotropy along the short-axes during contraction is similar in both the mitochondria and cell-edge measurements (Figs. 5 & 6) is consistent with these assumptions.

**Figure 7:**
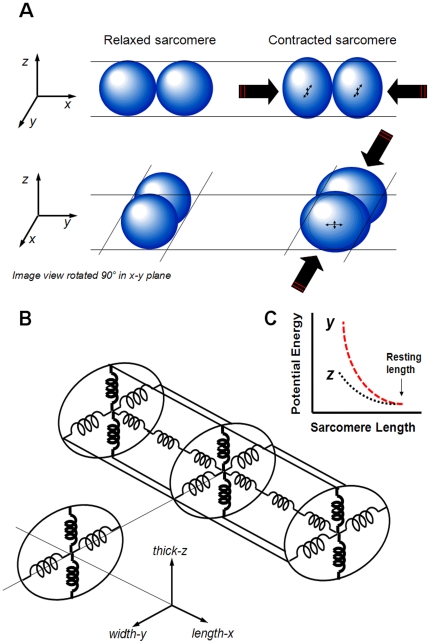
Scheme of mitochondrial 3D-deformation and proposed cytoskeleton mechanical analog. (A) Asymmetric radial expansion of mitochondria when compressed longitudinally by the sarcomeres during active contraction. While the mitochondria compress in the length-axis (***x***) they expand in the width-axis (***y***); however, the expansion in the thick-axis (***z***) is quite distinct from the width expansion. (B) An “equivalent spring” model of the cardiomyocyte cytoskeleton relevant to sarcomere lengths achieved during contraction, and capable of storing contractile energy during active cell shortening, then releasing this potential energy during relaxation as external work performed to relengthen the cell. The cytoskeleton is apparently stiffer in the cardiomyocyte thick-axis (shown in ***bold***) compared to the width-axis. (C) Based on a simple analog of Hookean springs, during cardiomyocyte contraction the elastic components of the cytoskeleton store more potential energy in the cell's *width*-axis compared to the *thick*-axis (see text for details).

Thus, the asymmetric expansion of mitochondria when compressed longitudinally during active contraction reveals that the stiffness of the cytoskeleton would likely have to be quite distinct along the two radial dimensions (summarized in Fig. 7). The cytoskeleton is apparently ∼3.5 times stiffer in the thick-axis compared to the width-axis (see below). Therefore, the measurement of 3D deformations is not only a tool to explore the mitochondria to better understand the energetic balance in the heart, but also to understand the force balance in the cytoskeleton. The molecular and structural nature of these passive load-bearing elements, responsible for the cardiomyocyte restoring force, remains to be defined.

Mitochondria in mammalian myocardium are organized radially (i.e., in the width- and thick-axes) between the contractile myofilaments (MF). de Tombe's laboratory showed that the MF lattice spacing is inversely proportional to sarcomere length [28], across the range of stretch from ∼1.9 to 2.25 µm sarcomere length, independent of the transition from diastole to systole [29]. Moreover, they found that the MF maintain essentially constant myofilament lattice *volume*, and propose that this likely persists during active contraction (although that aspect was not examined). Our findings, which apply to sarcomere lengths during active contraction (down to ∼1.8 µm at peak steady-state contraction), suggest that the coincident lateral spreading of *mitochondria* likely provides a radial “constrictive” force on the surrounding MF bundles, which by acting against the lateral constraints of some yet-to-be-defined set of stiff cytoskeletal elements bearing tension along the orthogonally-oriented short-axes (Fig. 7B), limits the tendency of MF to expand during contraction. Thus, this proposed physical role of the mitochondria could be important in constraining MF lattice spacing by limiting spreading near peak contraction, and thus facilitate optimization of MF overlap to augment peak active force development. It is notable that de Tombe also showed that the property of constant MF lattice *volume* during contraction in intact cardiomyocytes is lost after sarcolemmal permeabilization (as the normal constraints on MF lattice *spacing* are lost) [28]. We interpret this observation (in a minimalist way) to the inability of the cell to sustain the normally constant cytoplasmic volume during contraction, and to the loss of the normal radial force-balance in the cardiomyocyte.

### Potential energy storage considerations: Role of the cytoskeleton

PE can be stored in the cytoskeleton during contraction, which can be recovered during relaxation and cell elongation [1]. In isolated cardiomyocytes, the external loading and hence external work is negligible, but the PE stored in elastic cytoskeletal elements is largely recovered (minus viscous losses) during relaxation; therefore, the study of these 3D deformations is useful because there are implications for the role of various cytoskeletal elements in storing the PE that is important in relaxation. The apparent anisotropic behavior of the “transverse cytoskeleton” (or its equivalent, composed of elastic elements that provide transverse stiffness; Fig. 7B) suggests that the ability of the cytoskeleton to store PE to facilitate diastolic relaxation is different between the transverse axes.

For springs obeying Hooke's law, the displacement, ***x***, is in direct proportion to the force applied (and to a stiffness constant, ***k***), while the PE stored, being the integral of force over distance, increases as the *square* of the displacement, **PE = ½** ***kx*** **^2^**. As discussed above, it is reasonable to assume that the mitochondria exert and experience comparable vector forces along the width- and thick-axes when expanding in response to contractile compression, but because the displacement in the width-axis is ∼3.5 times that of the thick-axis, this means that the cytoskeleton is ∼3.5 stiffer in the *thick*- vs. *width*-axis (illustrated in Fig. 7B as thick vs thin springs, respectively). Thus, the ratio of PE stored (for comparable forces exerted) in the width- vs. thick-axis (Fig. 7C) is
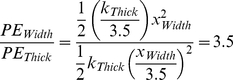
(5)


It has been determined that over the working range of the myofilaments and sarcomeres (typically above the slack sarcomere length) titin and collagen are the main elements that resist lengthening/stretch of the cardiomyocyte as well as the heart [30]. It has also been suggested that during contraction most of the restoring force-related PE is stored in elastic rather than viscoelastic elements [1], [31] and speculated that this elastic component may also be maintained by titin [32]. However, the true nature of those elements and structures responsible for transverse cardiomyocyte stiffness and storage of PE contributing to contractile relaxation have not been determined (see also comprehensive discussion of relevant literature in [33] **)**.

We can make an estimate of the potential importance of the recovery of this PE to cardiac relaxation. The potential energy stored in the cytoskeleton during the contraction cycle is approximately 10% of the total systolic work (estimated from the ratio of measured “diastolic” to “systolic” force-length work in single cardiomyocytes [34] and papillary muscle [35], [36]). Since the *ratio* of the work of atrial contraction compared to that of the ventricle is of a similar magnitude to that described above (estimated by comparing the pressure-volume work of the atria to that of the left ventricle [37]), we hypothesize that the contribution of the PE stored in the cardiomyocyte cytoskeleton to active relaxation and the early phase of ventricular filling (and hence, in turn to cardiac *systolic* function contributing to cardiac output [38], [39] due to Frank-Starling mechanisms) might be roughly comparable to that of atrial contraction during the latter phase of ventricular filling. The clinical observation that the atrial contraction contributes about 20% to left ventricular stroke volume [40], which is especially important for the performance of stiff hearts [41], [42] or at high beating rates when filling time is compromised, also suggests the potential importance of harnessing the PE stored in the cytoskeleton to the relaxation of the ventricle.

### Potential significance for aging and heart failure

During aging and heart failure the systolic and the diastolic properties of the heart become impaired [43]. Although much of the underlying pathophysiology of diastolic heart dysfunction remains obscure, nevertheless its clinical manifestations can play apparently significant roles in the functional decline during aging as well as after damage to the heart, e.g., after myocardial infarction, or potentially during medical conditions that affect the integrity and functions of proteins, such as may occur in diabetes. If the capacity of the cardiomyocyte cytoskeleton to store PE should become impaired, this could be manifest as a form of diastolic dysfunction, but there are few if any experimental means to examine this question functionally at the cellular level [44]. During aging the cardiomyocyte contraction duration increases substantially, while its amplitude and force development can remain functionally normal [45]. Additionally, there are changes in cardiomyocyte morphology in the aging heart, with the overall length increasing without significant proportional changes in the cell width and thickness [45], [46]. These superficial changes in geometry may also be accompanied by alterations in the cytoskeleton [47] which could adversely affect the stress-strain properties and thus potentially impair the ability to store PE during contraction, again contributing to diastolic dysfunction. We propose that further research into the nature of the cardiomyocyte cytoskeleton, in particular to understand the underlying cytoarchitecture and biophysical properties of those components that can store PE from contraction, and if/how these might change with aging, heart failure and other medical conditions, could be important in further elucidating the mechanisms of diastolic heart performance in health and disease.

### Mitochondrial fusion-fission events are not apparent

In certain cell types fusion and fission can be important for segregating dysfunctional or damaged mitochondria via mitophagy (prior to autophagy) and for normal function and homeostasis of the mitochondria (for review see [48]) including directing the production of ATP at certain subcellular sites (for review, see [49]). Fusion-fission has been detected in neonatal and cultured heart cells when they are actively dividing [50], [51] or in quiescent cells during cell growth. In neonatal cardiac cells, thapsigargin, which releases sarcoplasmic reticulum Ca^2+^ stores and increases intracellular Ca^2+^, leads to mitochondrial fission by inducing immediate fragmentation of many mitochondria [50]. It was also found that the mitochondrial inner membrane fusion protein, OPA1, was significantly decreased in the failing heart (rat model) without a change in the outer mitochondrial membrane proteins, Mfn1 and Mfn2, which are required to promote fusion [51]. The mitochondria in these *failing* rat hearts were small and fragmented compared with normal hearts, consistent with a decreased rate of fusion. However, based on the absence of data to the contrary, it is doubtful that mitochondrial fission and fusion occur amongst the sarcomeric mitochondria in adult cardiac myocytes from the *healthy* heart [52].

In healthy cardiomyocytes isolated from young adult rat hearts we found that the sarcomeric mitochondrial diameters returned to those at rest after transient sarcomere contraction. Therefore, under normal circumstances of cardiomyocyte contraction-relaxation cycling, the mitochondria arrayed between the sarcomeres remain discrete, deformable spheres whose plastic behavior reflects the mechanical forces around them, and, furthermore, there are no apparent fusion or fission events in this population under our experimental conditions. It should be noted, however, that the analytical method employed here, based on the Fourier transform, yields the average behavior of a large number of mitochondria, and rare events occurring in single mitochondria would likely not be detectable. We have also not examined the perinuclear pool of mitochondria, where it is possible that fusion-fission may yet be discovered to be playing important roles. Our results are in general agreement with Beraud et al [52] who found that under normal conditions fusion and fission of mitochondria are not detectable in the adult cardiomyocyte. Moreover, in *healthy* adult cardiomyocytes, the mitochondria behave as distinct entities (or as coupled pairs in the sarcomere) and apparently do not form electrically connected networks across multiple sarcomeres, since distinct individual mitochondria can be depolarized by photo-oxidative stress without depolarizing the neighbor mitochondrion [8], [52]. This taken together with the absence of direct evidence for fission-fusion suggests that in the healthy state a certain physical/functional insulating barrier exists between mitochondria in adjacent sarcomeres.

These results emphasize again the difference in the mitochondrial behavior of the isolated organelle vs. *in situ* conditions, and between various cell developmental stages, phenotypes and pathologies. While under normal conditions, fission and fusion were not observed under our experimental conditions, under pathological conditions (e.g., when the cytoskeletal properties may become pathologically damaged and impaired [47]), or at different stages of muscle development (when surprisingly dynamic movement of titin within and between sarcomeres has been observed in embryonic and neonatal cardiomyocytes [53]), organized complexes may readily become reorganized potentially allowing for mitochondrial rearrangements and fission and fusion to occur.

### Limitations

The mitochondrial 3D deformations were calculated based on actual measurements along two axes only, while assuming deformed-spherical/ellipsoidal geometry. Any deviation from the ideal ellipsoid (e.g., such as by forming a shape in compression more like a cube than that of a deformed-sphere/ellipsoid) will lead to an error in the calculated deformation in the thick-axis. We feel that this is not a significant limitation, as this deformation should still be qualitatively correct.

### Coda

In conclusion, the present results and analysis highlight the need for further investigations into the potential mechanosensitive nature of mitochondria as well as the structure and function of the cardiomyocyte cytoskeleton. This could lead to new insights into the regulation of mitochondrial function as well as to obtaining a better understanding of the dynamic force-balance inside cardiomyocytes and of changes in the spatial stiffness characteristics of the cytoskeleton that may accompany aging or pathological conditions that may govern diastolic performance of the heart.

## Materials and Methods

### Cells

Adult cardiac myocytes were isolated from Sprague-Dawley rats (male, 2–4 months old). All animal experiments were approved by the Institutional Animal Care and Use Committee of the Gerontology Research Center, National Institute on Aging, Intramural Research Program, NIH, protocol number 034-LCS-2013. Handling of animals and experimental procedures were conducted in accordance with NIH guidelines for animal care and use. Rats were anesthetized with sodium pentobarbital (200 mg/kg body weight), and all efforts were made to minimize suffering. Deep anesthesia was verified by checking absence of reflexes on repeated foot pinch and eye touch. After thoracotomy, hearts were rapidly excised and cannulated on a gravity driven Langendorff perfusion apparatus and perfused at 37°C with a solution containing (in mM): NaCl 120, KCl 5.4, NaH_2_PO_4_ 1.0, NaHCO_3_ 20, glucose 10, MgCl_2_ 1.6 (pH 7.2). An initial wash of approximately 3 min was followed by perfusion with buffer containing 4 mg/ml collagenase (Worthington) and 0.005 mg/ml protease. After 10-15 min the perfusion was stopped and the hearts were minced with scissors and dissociated with transfer pipettes. The cells were centrifuged (100 x *g*) for 1 min and suspended into the above solution fortified with 250 µM CaCl_2_. Finally, after purification by gravity sedimentation, the cells were suspended in HEPES buffer containing (in mM): NaCl 137, KCl 4.9, MgCl_2_ 1.6, NaH_2_PO_4_ 1.2, glucose 10, HEPES 20, and CaCl_2_ 1 (pH 7.3 with NaOH).

### Drugs

Where indicated, the cells were treated with 30 µM Diazoxide (Dz) (Sigma-Aldrich; 30 mM stock in DMSO) for 15 min. Cardiac myocytes were loaded with 125 nM tetramethylrhodamine methyl ester (TMRM) (Molecular Probes; 1 mM stock in DMSO) for at least 1.5 hours at room temperature. All other chemicals were obtained from Sigma-Aldrich.

### Confocal microscopy

Experiments were carried out at 36°C. Cells were imaged with a LSM-510 inverted confocal microscope using a 63x/1.4 N.A. oil immersion lens (Carl Zeiss). Transmitted optics line-scan images using 633 nm He-Ne laser illumination, 2048x1 pixels at 21.5 pixel/µm along the length (long) cell axis for 95.24 µm, and 21.5 pixel/µm along the width (short) cell axis for 17.85 µm, were recorded to detect long-axis sarcomere and mitochondrial dimensions, and short-axis mitochondrial dimensions, respectively (see definitions in Fig. 1A). Transmitted optics line-scan images using 633 nm He-Ne laser illumination, 512x1 pixels at 3.6 pixel/µm along the cell length- and width-axes were recorded to detect cell dimensions. In separate experiments, TMRM was excited with a 543 nm He-Ne laser, collecting fluorescence emission at wavelength > 560 nm, with the pinhole set to obtain a 1 µm optical slice, and zoom set at 21.5 pixel/µm. The images before and after treatment with Dz were obtained with zoom set at 21.5 pixel/µm.

### Experimental protocol

A freshly-isolated cell suspension was placed into a temperature-controlled perfusion chamber equipped with electrical field stimulation electrodes. During the data acquisition period, cells were at rest for 10 s, then stimulated at 1 Hz for 2.5 min, and rested again for 10 s. The analysis of mitochondrial, sarcomere, and cell dimensions during contraction was done during steady-state contractions. The voltage threshold for electrical stimulation was re-determined each experimental day and was set 10% higher than the voltage at which the majority of cells were contracting. An optical pulse synchronized with electrical stimulation was introduced into the first few seconds of the line-scan image to ensure that all data records were synchronized. The Dz group was superfused with HEPES buffer for 15 min followed by 15 min perfusion with 30 µM Dz in buffer. Only cardiomyocytes with the highest structural and functional integrity were utilized in these experiments to assess changes in sarcomere length and mitochondrial volume. Cells that did not exhibit a post-rest negative stair-case contractile pattern or reach steady state contractions, or that exhibited spontaneous calcium waves, were excluded.

### Analysis of data

Sarcomeres, as morphological and functional units of cardiomyocyte myofibrils, display a regular repeating band pattern when observed by light microscopy. Mitochondria arrayed in regularly ordered parallel rows surrounding contractile myofilaments create a unique beaded appearance that results from the regularity of the ultra-structure. Line- scanning along the long axis (Fig. 1B–C) reveals high-contrast dark stripes originating from the sarcomere Z-lines and gray stripes at the midline between Z-lines due (in part) to the boundary between mitochondria (organized at 2 per sarcomere). Line-scan imaging of *in situ* mitochondria was also performed along the width-axis (Fig. 1D) to identify mitochondrial deformation along this axis and to allow indirect assessment of mitochondrial deformation in the thick-axis under the assumption of constant mitochondrial volume. The periodicities of sarcomeres along the long axis and mitochondria along the long and short axes allowed us to analyze changes in the average dimensions of the sarcomeres and mitochondria. These two structures can be analyzed along the long axis of the cell by examining the amplitudes of the 1^st^ and 2^nd^ order peaks of the transmitted light optical contrast frequency spectrum resulting from the lattice patterns of sarcomere and mitochondrial structures, respectively (Fig. 2B–D, with the abscissa converted to µm, reciprocal frequency units). Fourier analysis (Matlab, Mathworks) of repeating gray level intensities of the transmitted optics line-scan image along the length-axis (Fig. 2A, recorded during 1 Hz electrical stimulation) reflects the spacing of the sarcomeres (Fig. 2B, upper left panel) and mitochondria (Fig. 2B, upper right panel). Fourier analysis of the line-scan image along the width-axis provides the short-axis mitochondrial dimension (Fig. 2B, lower panel). The analysis was done during both quiescent and contraction phases. Peak position in the frequency domain corresponds to average organelle dimension while the width of these peaks indicates the variation in size among sarcomeres or mitochondria in the cell at any moment in time. Due to the naturally lower number of repeating units along the cardiomyocyte width- compared to the length-axis and to a microscope acquisition limit of 2048 pixels, five to six independent line-scans across the width-axis, defined at the onset of the subsequent contractions, were concatenated into one long line scan prior to Fourier analysis (the scanning zoom was kept at 21.5 pixel/µm along the 17.85 µm line, but the net pixel number from which to extract information was increased from 2048 to 10240 or 12288). This method retains the modulus and frequency of principal peaks (which is essential for our method) and averages out variations in every digital level due to microscopic non-uniformity in optical contrast patterns during contraction between line-scans, but loses the phase information due to phase discontinuities between concatenated line-scans. To increase the spatial resolution during quiescent mode, a frame scan of the cell (scanning at 21.5 pixel/µm) was acquired and seven lines of this image in length or width were converted to one long line and analyzed. Fourier transform analysis permits spatial resolution of one cycle length, and experimental conditions were set such that spatial frequencies could be differentiated with precision down to a 2% change in a ∼1.95 µm structure (SL), and a 1% change in a ∼0.95 µm structure (mitochondrial dimension) along the cell length-axis for quiescent and contraction modes with a time resolution of 8 ms, and along the cell width-axis in the quiescent mode. To calculate deformation along the thickness axis (see definition in Fig. 1A), a 16th–order Butterworth band-pass filter selected for the desired frequency was applied for mitochondrial deformations along the length- and width-axes. Nonlinear regression fitting (Gauss-Newton algorithm) to the constant mitochondrial-volume model approximation (see Results) was applied (Matlab, Mathworks) to determine the ratio between mitochondrial deformations along the width- and thick-axes. The cell length and width were tracked by measuring the frequencies of the spectral peaks corresponding to the cell length and width, respectively. Nonlinear regression fitting (Gauss-Newton algorithm) to the constant cell-volume model approximation (see Results) was applied (Matlab, Mathworks) to determine the ratio between deformations in the cell width- and thick-axes.

*Statistics.* All experiments were performed with at least 9 cells in each control and Dz group. All data are presented as mean ± SEM. Comparisons were made using a one-way repeated measurements ANOVA test with p<0.05 taken to indicate statistical significance.
